# mTOR inhibition sensitizes T-ALL cells to Venetoclax through engagement of the integrated stress response

**DOI:** 10.1038/s41392-025-02368-8

**Published:** 2025-08-04

**Authors:** Loredana Urso, Irene Bertazzolo, Alberto Corradin, Micol Silic-Benussi, Sonia A. Minuzzo, Donna M. D’Agostino, Vincenzo Ciminale

**Affiliations:** 1 Veneto Institute of Oncology IOV – IRCCS, Padua, Italy; 2 University of Padova, Padova, Italy

**Keywords:** Cancer, Cancer therapy

**Dear Editor,**

Risk-adapted chemotherapy has significantly improved the clinical outcome of patients with T-cell acute lymphoblastic leukemia (T-ALL). However, approximately 20% of pediatric and 50% of adult T-ALL patients exhibit primary resistance or relapse to therapy.^1^ The identification of novel therapeutic avenues to treat these patients is thus a clinical priority.

T-ALL cells overexpress the anti-apoptotic protein BCL-2, making them potential targets for the BCL-2 inhibitor Venetoclax. However, while ETP-ALL cells are sensitive to Venetoclax, ‘typical’ T-ALL cells are resistant, due to the increased expression of BCL-xL, which is not targeted by Venetoclax.^2^

Our recent studies have centered on mTOR, a key pathway activated in T-ALL and associated with chemoresistance. We showed that pharmacologic or genetic inhibition of mTORC1 increased degradation of glucose-6-phosphate-dehydrogenase (G6PD), resulting in decreased levels of NADPH and increased ROS, which triggered apoptosis in T-ALL cells, but not in normal T-cells.^3^ mTORC1 inhibition also enhanced the killing effect of glucocorticoids,^3^ which act by increasing the expression of Bim, a proapoptotic BCL-2 family member. We thus hypothesized that mTORC1 inhibition might also enhance the effects of Venetoclax. We first tested the sensitivity of 6 T-ALL cell lines and 5 patient-derived xenografts (PDX) to Venetoclax at concentrations ranging from 1 nM to 1 µM. Figure 1a (‘Venetoclax-Everolimus’) shows that most T-ALL cell lines and PDX showed poor sensitivity to Venetoclax, measured as specific cell death (SCD). However, treatment with Venetoclax + Everolimus dramatically increased SCD in a subset of the cell lines and PDX, with TALL-1 and PDX13 showing the highest sensitivity, CEM and PDX19 cells showing the lowest sensitivity, and the other cell lines and PDX presenting intermediate responses. Bliss synergy analysis indicated that the interaction between Everolimus and Venetoclax was synergistic in TALL-1, Jurkat, PDX13 and PDX39 cells, suggesting that Everolimus unlocks sensitivity to Venetoclax in these cells (data not shown).

**Fig. 1:**
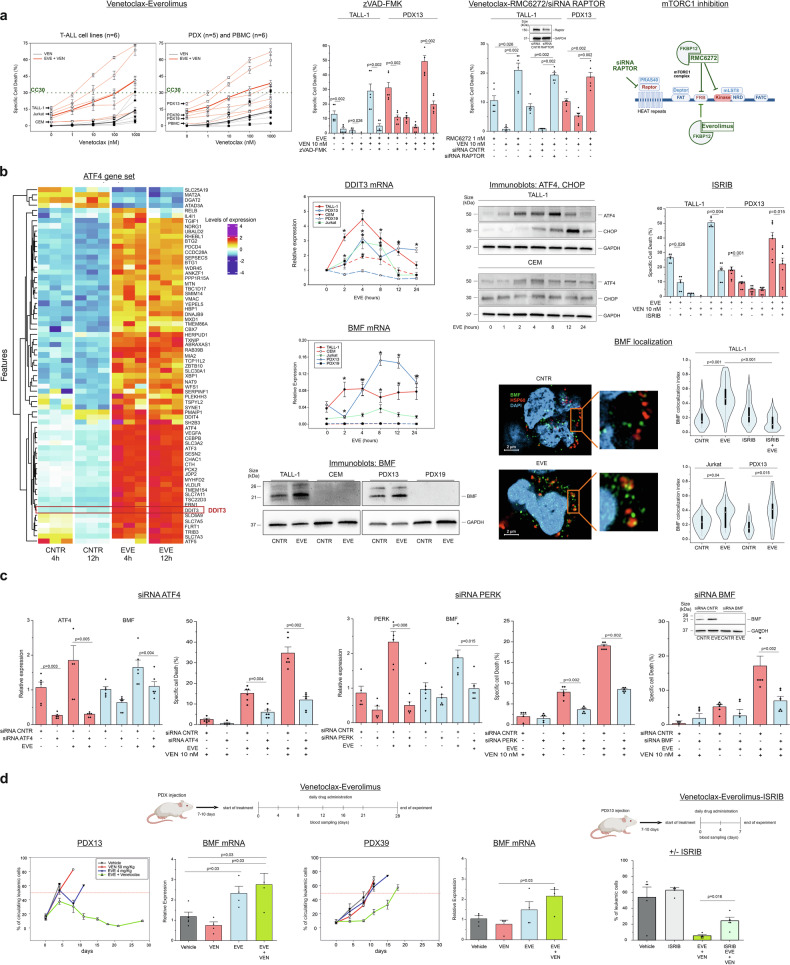
mTORC1 inhibition, Venetoclax sensitization, and the integrated stress response. **a** mTORC1 inhibition sensitizes T-ALL cells to Venetoclax. ‘Venetoclax-Everolimus’ shows specific cell death (SCD, see Supplementary Material) of T-ALL cell lines, T-ALL PDX, and control PBMC after 24 h’ treatment with the indicated concentrations of Venetoclax alone (grey lines) or with Venetoclax plus 10 µM Everolimus (EVE, red lines). Thin lines indicate results for individual cell lines or PDX (means ± SE from 3 experiments performed in triplicate), and thick lines indicate means for all cell lines or PDX. The first data points on the red lines show SCD induced by 10 µM Everolimus alone. The dotted green line indicates 30% specific cell death (SCD) to facilitate the direct visualization of the 30% cytotoxic concentrations (CC30). Arrows indicate the best- (TALL-1 and PDX13) and worst-responding (CEM and PDX19) cell lines/PDX, as well as PBMC (SCD ± SE of PBMC from 6 healthy donors). Differences between Everolimus-treated vs. vehicle-treated, Venetoclax+Everolimus-treated vs. vehicle-treated and Venetoclax+Everolimus-treated vs. Venetoclax-treated cells were statistically significant (*p* < 0.05, Mann Whitney test). ‘zVAD-FMK’ shows the effect of the pan-caspase inhibitor z-VAD-FMK on SCD induced by the drug combinations. ‘Venetoclax-RMC6272/siRNA RAPTOR’ shows SCD of TALL-1 cells after 48 h’ treatment with the bisteric mTORC1 inhibitor RMC6272 (1 nM) alone or plus Venetoclax (10 nM) and SCD of TALL-1 cells after electroporation with negative control siRNA (CNTR) or RAPTOR-siRNA followed by treatment with vehicle or Venetoclax (10 nM). The immunoblot shows levels of Raptor in a representative silencing experiment; GAPDH served as a loading control. ‘mTORC1 inhibition’ shows the components of the mTORC1 complex targeted by the different inhibitors used in the experiments. The scheme was drawn using BioRender (https://app.biorender.com/illustrations/680a01dde8cce2497b4a907c). **b** Engagement of the Integrated Stress Response (ISR) and expression of BMF following mTORC1 inhibition. The heatmap ‘ATF4 gene set’ shows differentially expressed genes included in the ATF4 gene set (see Supplementary Material) identified in TALL-1 cells treated with 10 µM Everolimus or vehicle for 4 or 12 hours. Normalized gene counts were Z-score-transformed and a custom color palette was applied to highlight differences in expression. ‘DDIT3 mRNA’ shows qRT-PCR analysis of the DDIT3 mRNA (coding for CHOP) in TALL-1, Jurkat, CEM, PDX13, and PDX19 cells treated with 10 µM Everolimus for 0, 2, 4, 8, 12, and 24 h. Asterisks indicate statistically significant differences between individual time points and matching time 0 values (means ± SE from three independent experiments performed in duplicate). ‘Immunoblots: ATF4, CHOP’ shows ATF4, CHOP, and GAPDH protein levels in TALL-1 and CEM cells treated with 10 µM Everolimus for 0, 1, 2, 4, 8, 12, or 24 h. ‘ISRIB’ shows specific cell death in TALL-1 and PDX13 cells pre-treated with 25 nM ISRIB for 2 h before treatment with Everolimus and/or Venetoclax for 24 h (means ± SE from three independent experiments performed in duplicate). ‘BMF mRNA’ shows qRT-PCR analysis of the BMF mRNA in TALL-1, Jurkat, CEM, PDX13 and PDX19 cells treated with 10 µM Everolimus for 0, 2, 4, 8, 12, or 24 h. Shown are mean 2^-ΔCt^ values ± SE from three independent experiments performed in duplicate. Asterisks indicate statistically significant differences between individual time points and matching time 0 values. Curves for CEM and PDX19 cells approach zero and are overlapping. ‘Immunoblots: BMF’ shows BMF and GAPDH proteins detected in TALL-1, CEM, PDX13, and PDX19 cells treated with vehicle or 10 µM Everolimus for 24 h. ‘BMF localization’ shows the effects of 24 h’ treatment with Everolimus on the intracellular distribution of BMF. Images show BMF (green), the mitochondrial protein HSP60 (red) and DAPI (blue, to identify nuclei) in TALL-1 cells. The violin plots show the median weighted colocalization index of 20 (TALL-1), 15 (Jurkat) and 8 (PDX13) randomly-selected fields (with an average of 3 cells per field) for each treatment group, where the weighted colocalization index in each field was evaluated as the mean of colocalization in adjacent stacks on the z-axis. The top violin plot includes results obtained for TALL-1 cells pretreated with 25 nM ISRIB for 2 hours. **c** Functional validation of the PERK-ATF4-BMF axis. TALL-1 cells were electroporated with siRNA targeting ATF4, PERK or BMF or control siRNA and then treated with 10 μM Everolimus, 10 nM Venetoclax or with both drugs for the treatment times indicated in the Supplementary Material. Shown are results of qRT-PCR and specific cell death assays (means ± SE from 3 experiments performed in duplicate), and an immunoblot to detect BMF protein. **d** Everolimus and Venetoclax reduce leukemic burden in vivo. ‘Venetoclax-Everolimus’ summarizes the in vivo experiment in which PDX-inoculated NOD/SCID mice (5 per experimental group) were treated with vehicle, Venetoclax, Everolimus, or both drugs as described in the Supplementary Material. ‘PDX13’ and ‘PDX39’ graphs show mean percentages ± SE of circulating CD5/CD7-positive leukemic cells measured at the indicated time points. ‘BMF mRNA’ graphs show mean relative expression of BMF mRNA ± SE in PDX13 and PDX39 leukemic cells isolated from spleens when circulating leukemic cells exceeded 50% of total PBMC or at the end of the experiment (28 days). ‘Venetoclax-Everolimus-ISRIB’ summarizes the in vivo experiment with PDX13-inoculated NOD/SCID mice treated with vehicle, ISRIB, Venetoclax plus Everolimus, or Venetoclax plus Everolimus plus ISRIB (see Supplementary Material). ‘−/+ISRIB’ shows mean percentages of CD5/CD7-positive leukemic cells ± SE in peripheral blood of the mice after 7 days’ treatment. For all experiments shown in Fig. 1, statistically significant differences (*p* < 0.05) were determined using the Mann-Whitney or Wilcoxon Signed Rank test (see Supplementary Material)

Notably, PBMCs from healthy donors were less sensitive to the drug combination compared to T-ALL cells (Fig. 1a). The observation that cell death was reduced by the pan-caspase inhibitor z-VAD-FMK (Fig. 1a, ‘zVAD-FMK’) implicated apoptosis as the death mechanism.

The effects of Everolimus were recapitulated by blockade of mTORC1 with the “bisteric” inhibitor RMC6272 or through siRNA-mediated silencing of Raptor, an essential component of the mTORC1 complex (Fig. 1a, ‘Venetoclax-RMC6272/siRNA RAPTOR’), thus supporting a key role of this pathway in enhancing the effects of Venetoclax.

To investigate the mechanism by which Everolimus sensitizes T-ALL cells to Venetoclax, we measured the levels of BCL-2, BCL-xL, and MCL1 and found no changes in response to Everolimus (data not shown). The response to Everolimus also did not correlate with alterations commonly observed in T-ALL, such as mutations in *NOTCH1* or *TP53*, or loss of PTEN (data not shown).

To explore the downstream effects of mTORC1 inhibition, we performed RNAseq on TALL-1 and CEM cells (representative of a high and poor response, respectively) following Everolimus treatment. Principal component analysis (PCA) revealed a stark separation of Everolimus-treated from vehicle-treated samples. Many of the genes differentially expressed in TALL-1 cells showing the highest weights in the PC1 projection belonged to gene ontology terms related to response to cellular stress (data not shown). Gene set enrichment analysis (GSEA) indicated that the gene set for ATF4, the master transcriptional regulator of the Integrated Stress Response (ISR)^4^ (see Supplementary Material), was enriched in the “responder” TALL-1 cells (NES = 2.30; FDR q-value = 0.003), but not in non-responder CEM cells (NES = 0.96; FDR q-value = 0.73).

Current knowledge suggests that the ISR can trigger either cell death or survival depending on the amplitude and duration of the stress.^4^ To investigate the temporal trajectories of ISR engagement, we performed RNAseq analysis on TALL-1 cells treated with Everolimus for 4 or 12 hours. Results showed that Everolimus induced a rapid and sustained upregulation of a subset of ATF4-related genes that included *DDIT3*, which codes for CHOP, a transcription factor that controls the pro-apoptotic arm of the ISR^4^ (see heatmap in Fig. 1b). Quantitative RT-PCR (qRT-PCR) to measure the DDIT3 mRNA after 0, 2, 4, 8, 12, and 24 hours’ treatment with Everolimus revealed a robust and sustained increase in DDIT3 expression in TALL-1, PDX13 and Jurkat cells, but not in CEM or PDX19 cells (Fig. 1b, ‘DDIT3 mRNA’). Immunoblots to detect the ATF4 and CHOP proteins showed that both proteins were more robustly induced in TALL-1 compared to CEM cells (Fig. 1b, ’Immunoblots: ATF4, CHOP’). Consistent with these findings, pharmacological inhibition of the ISR with ISRIB significantly blunted the cell death induced by Everolimus and Venetoclax (Fig. 1b, ‘ISRIB’).

To investigate the impact of ISR engagement on apoptosis, we analyzed RNAseq data from TALL-1 cells using a custom gene set that comprised 18 apoptosis-related genes (see Supplementary Material). Results revealed upregulation of BMF, a pro-apoptotic BCL2 family member, in Everolimus-treated cells (data not shown). qRT-PCR analysis revealed significant differences in BMF mRNA levels between “responder” cells (TALL-1, Jurkat and PDX13) and non-responders (CEM and PDX19, *p* < 0.005). Everolimus treatment resulted in a sustained increase in BMF mRNA levels in TALL-1 and PDX13 cells, a more modest increase in Jurkat cells, and no change in CEM and PDX19 cells (Fig. 1b, ‘BMF mRNA’). Differences in BMF expression were confirmed by immunoblot analysis (Fig. 1b, ‘Immunoblots: BMF’). Furthermore, confocal microscopy analysis of TALL-1, Jurkat and PDX13 cells showed that Everolimus increased the targeting of BMF to mitochondria, a key step required to trigger its proapoptotic function^5^; this effect was blocked by ISRIB (Fig. 1b, ‘BMF localization’).

The functional relevance of the ISR was confirmed by siRNA-mediated knockdown of ATF4 in TALL-1 cells, which blunted the effects of Everolimus on BMF expression; ATF4 knockdown also reduced death induced by Everolimus, Venetoclax and the drug combination (Fig. 1c, ‘siRNA ATF4’). Cell death induced by Everolimus or Everolimus+Venetoclax was also blunted by knockdown of PERK, one of the apical kinases that engage the ISR^4^ (Fig. 1c, ‘siRNA PERK’). Importantly, knockdown of BMF also reduced the killing of TALL-1 cells by Everolimus and Everolimus + Venetoclax (Fig. 1c, ‘siRNA BMF’). These observations support the link between mTORC1 inhibition, triggering of the ISR, upregulation of BMF, and apoptotic death.

The efficacy of Everolimus/Venetoclax in a pre-clinical setting was evaluated by injecting PDX13, PDX39, and PDX19 cells into NOD/SCID mice followed by treatments indicated in Fig. 1d. Results showed that the combination of Everolimus and Venetoclax resulted in a sustained reduction of the leukemic burden in PDX13-inoculated mice compared to single-agent treatments, while in PDX39-inoculated mice the combination initially reduced leukemic cells, but relapse occurred after 10 days. Consistent with our in vitro data, PDX19 was insensitive to this drug combination (data not shown). The effect of Everolimus+Venetoclax on PDX13 and PDX39 in vivo was accompanied by upregulation of BMF mRNA (Fig. 1d, ‘BMF mRNA’). A companion experiment showed that the effect of Venetoclax plus Everolimus was blunted by ISRIB, indicating dependence on the ISR in vivo (Fig. 1d, ‘Venetoclax-Everolimus-ISRIB’).

In summary, these findings provide insight into the links between mTORC1 and the ISR and furnish proof-of-concept evidence for mTORC1 inhibition and sustained ISR activation as a viable strategy to overcome resistance to Venetoclax in a subset of T-ALL. Expression of BMF, a key mediator of this process, could predict response to this combination therapy.

## Supplementary information

Supplementary information

## Data Availability

The RNAseq data elaborated in this study are deposited in the NCBI Gene Expression Omnibus and are accessible through GEO series accession numbers GSE273650 and GSE225751.
