# Supplementary material for: mTOR inhibition sensitizes T-ALL cells to Venetoclax through engagement of the integrated stress response
Source: Signal Transduct Target Ther. 2025 Aug 4;10:246. doi: 10.1038/s41392-025-02368-8 (PMC12319092; doi:10.1038/s41392-025-02368-8)
Supplement: Supplementary file 1 — Supplementary information [file 41392_2025_2368_MOESM1_ESM.docx]

**Supplementary Material for**

**mTOR inhibition sensitizes T-ALL cells to Venetoclax through engagement of the integrated stress response**

Loredana Urso*, Irene Bertazzolo, Alberto Corradin, Micol Silic-Benussi, Sonia A. Minuzzo, Donna M. D’Agostino, and Vincenzo Ciminale^*^

*Correspondence to: [loredana.urso@iov.veneto.it](mailto:loredana.urso@iov.veneto.it) and [v.ciminale@unipd.it](mailto:v.ciminale@unipd.it)

**This file includes:**

Materials and Methods

**Materials and Methods**

**T-ALL cell lines and PDX.**The T-ALL-derived cell lines CEM, DND41, Jurkat, MOLT-3, PF382 and TALL-1 were cultured in RPMI 1640 medium (Euroclone) supplemented with 10% fetal bovine serum (Sial), 2 mM L-glutamine, 100 units/mL penicillin, and 20 units/mL streptomycin (complete RPMI). Patient-derived xenograft cells (PDX) were derived from primary leukemia cells of pediatric T-ALL patients and propagated in NOD/SCID mice as previously described^1,2^. PDX cells were re-isolated from the spleens of the host mice and either used for short-term *in vitro* drug testing or re-inoculated into NOD/SCID mice for *in vivo* experiments (see below). The PDX cells used here are not adapted to growth *in vitro* and can be cultured for a limited period (2-3 days). Peripheral blood mononuclear cells (PBMCs) from healthy donors were isolated by Ficoll-Paque Plus (GE Healthcare) gradient centrifugation following the manufacturer's protocol and cultured in complete RPMI for up to 48 hours.

***In vitro* drug treatments.** Cells (1x10^6^/ml) were treated with vehicle (0.1% ethanol), Everolimus (Selleck Chemicals), Venetoclax (Medchem Express), RMC6272 (Medchem Express), or drug combinations at the concentrations and incubation times indicated in the figure, and analyzed as described below. In some experiments, the cells were pre-incubated with 25 nM ISRIB (Sigma-Aldrich) or 50 µM Z-VAD-FMK (Selleck Chemicals) for 2 hours prior to addition of Everolimus and/or Venetoclax.

**Cell death assays.** Cell death was assessed by measuring the uptake of propidium iodide (PI, Sigma-Aldrich). Aliquots of cells (2x10^5^) were suspended in 200 µl of complete RPMI containing 400 ng PI. After 10 minutes’ incubation at room temperature, the cells were analyzed by flow cytometry using a FACSCelesta flow cytometer (BD Bioscience) and DIVA software. Data from 10,000 ungated events were used to calculate specific cell death (SCD) using the following formula: [(% of dead cells in drug-treated sample - % of dead cells in vehicle-treated control) / (100 - % of dead cells in mock-treated control)] x 100.

**RNAseq analysis of Everolimus-treated cell lines.** RNAseq analyses of TALL-1 and CEM cells after treatment with 10 µM Everolimus or drug vehicle was carried out as described^3,4^. A second RNAseq analysis was performed to explore the kinetics of the response of TALL-1 cells treated with 10 µM Everolimus or vehicle for 4 and 12 hours. Total RNA was processed at Personal Genomics (Sol S.p.A., Monza) for ribo-depletion (RD)-based RNAseq library preparation and RNAseq using an Illumina platform. Approximately 40 million reads per sample were obtained.

RNAseq data were analyzed with bioinformatic tools assembled in a recently developed pipeline^5^. Raw gene counts were normalized and tested (Wald statistical test and Benjamini-Hochberg multiple testing correction, p-adj<0.05) with the DeSeq2 algorithm^6^. Differentially expressed features were selected among statistically significant sequences with relevant fold-change values, and coding genes were retained for further analyses. The ATF4 gene set, described in ref.^7^, includes 794 genes induced in response to tunicamycin in wild type mouse embryo fibroblasts (MEFs) but not in ATF4-KO MEFs. Sixty-nine genes were identified as differentially expressed in TALL-1 cells treated with Everolimus compared to the control: downregulated genes comprised SLC25A29, MAT2A, DGAT2, ATAD3A; upregulated genes included RELB, IL4I1, TGIF1, NDRG1, UBALD2, RHEBL1, BTG2, PDCD4, CCDC28A, SEPSECS, BTG1, WDR45, ANKZF1, PPP1R15A, MNT, TBC1D17,SMIM14, VMAC , YEPEL5, HBP1, DNAJB9, MXD1, TMEM86A, CBX7, HERPUD1, TXNIP, ABRAXAS1, RAB39B, MIA2, TCP11L2, ZBTB10, SLC30A11, XBP1, NAT9, WFS1, SERPINF1, PLEKHH3, TSPYL2, SYNE1, PMAIP1, DDIT4, SH2B3, ATF4, VEGFA, CEBPB, SLC3A2, ATF3, SESN2, CHAC1, CTH, PCK2, JPD2, MTHFD2, VLDLR, TMCM154, SLC7A11, TSC22D3, ERN1, DDIT3, SLC6A3, SLC7A5, FLRT1, TRIB3, SLC7A3, ATF5. A proapoptotic custom gene set included the following 18 genes: BAX, BAK1, BOK, BID, BCL2L11, BCL2L13, BBC3, PMAIP1, HRK, BIK, BAD, BMF, BNIP3L, BNIP3, TNFSF10, TNFRSF10B, TNFRSF10A, TNFRSF10C.

**RNA extraction and quantitative RT-PCR (qRT-PCR).** Total RNA was isolated and reverse-transcribed as described^3^. Aliquots of the resulting cDNA were PCR-amplified by using SYBR Green Master Mix (Roche) and the following primers: β2-microglobulin- forward: GCATTCCTGAAGCTGACAGC; β2-microglobulin-reverse: CAATGTCGGATGGATGAAACC; DDIT3-forward: TTCTCTGGCTTGGCTGACTG; DDIT3-reverse: TCCTCCTCTTCCTCCTGAGC; BMF-forward: ATGCTGGCTATCGGCTTCCT; BMF-reverse: GATCTGCCACCACACACGAT. The PCR reactions were performed in a LightCycler 480 thermal cycler (Roche) according to the manufacturer's protocol. Relative expression values were calculated using the 2^-ΔΔCt^ method^8^, with β2-microglobulin serving as a housekeeping gene for the calculations. Changes in DDIT3 mRNA levels at different treatment timepoints (Fig. 1b ‘DDIT3 mRNA’) were calculated by scaling against the expression levels measured at the beginning of the experiment (T0). The graph ‘BMF mRNA’ shows 2^-ΔCt^ values to highlight differences in BMF mRNA levels in the cell lines and PDX.

**Immunoblotting.** Cells were lysed in cell disruption buffer (25 mM Tris-HCl, pH 7.4, 150 mM EDTA, 1% Nonidet-P40, 5% glycerol) containing inhibitors of phosphatases and proteases (PhosphoSTOP and Complete, Roche). The protein concentration was determined with the Coomassie Plus Protein Assay reagent (Thermo Fisher Scientific). Twenty-five µg of protein were separated by SDS/PAGE in 4-15% mini-PROTEAN TGX Stain-Free precast gradient gels (BioRad) and then transferred to nitrocellulose membranes using a Trans-Blot Turbo apparatus (BioRad). The membranes were saturated with 5% bovine serum albumin (BSA) in Tris-buffered saline (TBS) (50 mM Tris-HCl, pH 7.5, 150 mM NaCl)-0.01% Tween-20 and then incubated overnight in mouse/rabbit Purity Western Blot Detection System reagent (Vilber Lourmat). Primary antibodies included rabbit anti-BMF (Cell Signaling Technology, 1:1500), rabbit anti-Raptor (Cell Signaling Technology, 1:1000), rabbit anti-ATF4 (Proteintech, 1:1000), rabbit anti-CHOP (Proteintech, 1:1000), and rabbit anti-GAPDH (GeneTex, 1:10,000). Membranes were washed twice and chemiluminescent signals were detected using LiteAblotTURBO (EuroClone) and a Cambridge UVITEC imaging system.

**siRNA-mediated knockdown experiments.** TALL-1 cells (5x10^6^) were mixed with 312.5 pmol of Silencer Select siRNA (ATF4 #S1404, BMF #S40385, EIF2AK3-PERK #S18102, or Silencer Negative Control 1 #4390843, Thermo Fisher Scientific) or ON-TARGET plus SMARTpool siRNA (RAPTOR # 004107000005, or ON-TARGET PLUS Non targeting pool #0018101005, Dharmacon), and electroporated with a Neon transfection system (Thermo Fisher Scientific) using a single 1410 V-30 msec pulse. Cells electroporated with PERK and ATF4 siRNAs were cultured for 24 hours and then treated with Everolimus and/or Venetoclax for 4 hours for qRT-PCR, or for 24 hours for SCD analyses. Cells electroporated with the BMF siRNA were cultured for 48 hours and then treated with Everolimus and/or Venetoclax for 24 hours for immunoblot and SCD analyses. Cells electroporated with Raptor siRNAs were cultured for 48 hours and then treated with Everolimus and/or Venetoclax for 24- or 48 hours for immunoblot and SCD analyses, respectively.

**Immunofluorescence analysis to detect BMF and HSP60.** Cells were fixed for 15 minutes with 4% formaldehyde (Thermo Fisher Scientific), permeabilized for 10 minutes with TBS-0.01% NP40, and incubated for 1 hour at 37°C with rabbit anti-BMF antibody (1:100, Cell Signaling Technology) and chicken anti-HSP60 antibody (1:500, Novus Biological) followed by Alexa Fluor 488-conjugated goat anti-rabbit and Alexa Fluor 555-conjugated goat anti-chicken antibodies (Thermo Fisher Scientific, 1:1000) for 45 minutes at 37°C; samples were rinsed in TBS and centrifuged after each incubation. Samples were mounted with an anti-fade reagent containing DAPI (Prolong Diamond, Molecular Probes) to visualize nuclei. Images were obtained with a Zeiss Airyscan LSM900 confocal microscope. Mitochondrial localization of BMF was quantitated by calculating the weighted colocalization index of BMF and HSP60 using ZenBlue software.

***In vivo* experiments using T-ALL PDX in NOD/SCID mice.** All procedures involving animals and their care were authorized by the ethics committees of the University of Padova and the Italian Ministry of Health (animal welfare committee approval no. 183/2022 PR) in compliance with the ARRIVE guidelines and in accordance with European Union directives (86/609/EEC and 2010/63/EU). *In vivo* experiments were performed on NOD/SCID mice housed in a specific pathogen-free (SPF) facility and cared for as described previously^3^. The experiments were performed with 3 PDX that differed in their *in vitro* sensitivity to Everolimus plus Venetoclax (PDX13, PDX39 and PDX19). 6-8-week-old female mice, (weighing ⁓20 g) were inoculated with 5x10^6^ PDX cells by injection into the tail vein. Seven to 10 days later, when the percentage of circulating leukemic cells reached 5-10% of the total PBMC, mice were randomized, assigned in blind to the experimental groups, tagged for identification, and treated daily by oral gavage with either 30% polyethylene glycol (PEG)/70% H_2_O (vehicle control), Venetoclax (50 mg/kg), Everolimus (4 mg/kg), Venetoclax (50 mg/kg) + Everolimus (4 mg/kg), ISRIB (2.5 mg/Kg), or ISRIB (2.5 mg/Kg) + Everolimus (4 mg/kg) + Venetoclax (50 mg/Kg). Blood samples were obtained from the submandibular plexus. Mice were sacrificed when circulating tumor cells exceeded 50% of total PBMC. Euthanasia was carried out by cervical dislocation.

**Statistical analysis and graphics.** Differences between treatment groups were analyzed using the Mann-Whitney rank sum test or the Wilcoxon Signed Rank test for matched samples (Figure 1b ‘DDIT3 mRNA’ and Figure 1c ’BMF mRNA’) using SigmaPlot software. Statistically significant differences (p < 0.05) are indicated in the figure. Graphs were generated using SigmaPlot 15.0 (Systat Software Inc.) or OriginPro 2024 (Origin Lab Corporation).  The figure was generated using CorelDraw. The graphical scheme was created with [BioRender.com](https://biorender.com/).

**REFERENCES**

1 Agnusdei, V. *et al.* Therapeutic antibody targeting of Notch1 in T-acute lymphoblastic leukemia xenografts. *Leukemia* **28**, 278-288 (2014).

2 Minuzzo, S. *et al.* Targeting NOTCH1 in combination with antimetabolite drugs prolongs life span in relapsed pediatric and adult T-acute lymphoblastic leukemia xenografts. *Exp. Hematol. Oncol.* **12**, 76 (2023).

3 Silic-Benussi, M. *et al.* mTOR inhibition downregulates glucose-6-phosphate dehydrogenase and induces ROS-dependent death in T-cell acute lymphoblastic leukemia cells. *Redox Biol.* **51,**  102268 (2022).

4 Silic-Benussi, M. *et al.* Repurposing Verapamil to Enhance Killing of T-ALL Cells by the mTOR Inhibitor Everolimus. *Antioxidants*  **12**, 625 (2023).

5 Corradin, A. *et al.* Gene Set-Focused Analysis of RNA-Seq Data with MIEP (Make-It-Easy-Pipeline) in Lecture Notes in Bioinformatics, Vol. 15276 (eds. Cerulo, L. et al.), 58-68 (Springer, 2025).

6 Love, M., Huber, W. & Anders, S. Moderated estimation of fold change and dispersion for RNA-seq data with DESeq2. *Genome Biol.* **15**, 550 (2014).

7 Torrence, M. *et al.* The mTORC1-mediated activation of ATF4 promotes protein and glutathione synthesis downstream of growth signals. *eLife* **10**, e63326 (2021).

8 Livak, K. & Schmittgen, T. Analysis of relative gene expression data using real-time quantitative PCR and the 2(-Delta Delta C(T)) Method. *Methods* **25**, 402-408 (2001).
